# Trends in mortality inequalities in an urban area: the influence of immigration

**DOI:** 10.1186/s12939-019-0939-9

**Published:** 2019-02-26

**Authors:** Maica Rodríguez-Sanz, Mercè Gotsens, Marc Marí dell’Olmo, Carme Borrell

**Affiliations:** 1 Agència de Salut Pública de Barcelona, Lesseps, 1, 08023 Barcelona, Spain; 2 Institut d’Investigacio Biomedica (IIB Sant Pau), Barcelona, Spain; 3 CIBER Epidemiología y Salud Pública (CIBERESP), Barcelona, Spain; 4 Universitat Pompeu Fabra, Barcelona, Barcelona, Spain

**Keywords:** Health equity, Epidemiology

## Abstract

**Background:**

Nearly 20% of the population in Barcelona is foreign-born and this percentage rises to up to 40% in some neighborhoods. Consequently, migration health patterns may play an important role in trends in socioeconomic geographical inequalities in mortality. The objective of this study was to analyze the trend in socioeconomic inequalities in mortality between neighborhoods in Barcelona during the period 2001–2012 in the foreign-born and Spanish-born population.

**Methods:**

Repeated cross-sectional design of the population aged 25–64 years in Barcelona between 2001 and 2012. Hierarchical data consisted of yearly mortality linked-population. The variables analyzed were age, sex, education, and country of birth (Spanish-born, foreign-born), neighborhood of residence, and the socioeconomic level of the neighborhoods using quartiles of unemployment rates. Age-standardized mortality rates were estimated, and mixed Poisson regressions were applied using generalized linear mixed models, including two random effects to consider the intracorrelation within neighborhoods and across years.

**Results:**

The number of foreign-born residents aged 25–64 increased notably in disadvantaged neighborhoods. Approximately 9% of premature deaths occurred in foreign-born individuals. Premature mortality rates were higher in disadvantaged neighborhoods and in the Spanish-born population in all periods. Despite the stabilized socioeconomic inequalities in mortality in the Spanish-born population, no inequalities were found between neighborhoods in foreign-born men and women.

**Conclusions:**

Evidence of the ‘healthy migrant’ effect in mortality and socioeconomic inequalities in mortality was found in Barcelona, which seems to alter the distribution of mortality through time and space, related to the low levels of premature mortality and the selective residence of immigrants in socioeconomically disadvantaged neighborhoods.

## Introduction

More than half the world’s population lives in urban areas, reaching 70% in high-income countries. This trend is increasing mainly because populations migrate to cities seeking better working and living conditions [1]. Nonetheless, cities have certain risks, where social inequalities also exist, as there are socioeconomically disadvantaged neighborhoods or areas where the most vulnerable populations tend to concentrate, leading to poorer health among their residents [2, 3].

During the 1980s, the foreign-born population rose in some European countries and, mainly after 2000, it increased in most western and central European countries, although this migration flow decreased after the start of the global economic recession in 2008 [4]. In Spain, the migrant population rapidly grew from 4% of the population in 2000 to 12% in 2009 [5], whereas in Barcelona in 2009 the foreign-born population represented 19% of residents, with seven out of ten coming from countries such as those of Latin America, Morocco, China and Pakistan [6, 7]. The growth of immigration is usually unequal across space, with some residential segregation patterns being reproduced by selective migration processes that sort immigrants into socioeconomically disadvantaged neighborhoods. This is true of Barcelona, where the center of the city, the starting point for new arrivals, and some peripheral neighborhoods play host to more than 40% of foreign-born residents [6].

Previous research has provided support for the concept of “migrant selectivity”, as migrants are often, at least initially, younger and healthier than non-migrants in their host countries, a phenomenon known as the “healthy migrant effect”. In addition, migrants tend to have significantly lower mortality than persons in their host countries, because immigrants who are ill may return to their native countries for treatment or to die, a process called “salmon bias” [8]. Studies on migrant mortality in the European context have examined different issues. Some of them have reported that overall and premature mortality was lower for the foreign-born than the local born population, independently of individual socioeconomic characteristics [9, 10]. In addition, some studies analyzed how this pattern was mediated by sex, age and country of origin [11–17] and depended on the specific cause of death [11, 12, 15–18]. Few studies have analyzed the association between immigrant mortality and greater length of residence [13, 19, 20]. A study has suggested that immigrant mortality may be influenced by different integration policy contexts in host countries [21].

On the other hand, some studies have found that population growth and selective migration could be responsible for a change in geographical mortality inequalities between the least and most deprived areas [22]. One study that analyzed 250 regions in 26 European countries found that areas with large population gains through migration were more likely to have low death rates, independently of area socioeconomic status [23]. Narrowing inequalities have been described in the UK, with less deprived areas experiencing population gain and these areas having lower premature mortality rates, notably in London [24]. In contrast, in France [25], reductions in mortality were found in deprived areas showing population growth, related to new residential areas and/or an influx of a wealthier population. In Barcelona, a recent study found that the reduction in inequalities in mortality between neighborhoods was related to an increase in the foreign-born population in disadvantaged neighborhoods [26]. Moreover, the economic recession, initiated in 2008, has impacted mortality and its inequalities [27, 28].

Despite a rapid increase in immigration from low-income countries, studies on immigrant mortality in Spain are scarce. Immigration patterns may play an important role in trends in socioeconomic geographical inequalities in mortality, especially in places such as Barcelona where there is selective residential immigration [6]. The objective of this study was therefore to analyze the trend in socioeconomic inequalities in mortality in the foreign-born and Spanish-born population between neighborhoods in Barcelona during the period 2001–2012.

## Data and methods

This study used repeated cross-sectional data from the population aged 25–64 years resident in Barcelona between 2001 and 2012. We studied this group of age in order to include population with education finished (more than 25 years of age) and taking into account that immigrant population is younger than 65 years of age. Moreover, premature mortality, which implies deaths that occur prematurely, usually includes mortality under 65–70 years of age [29]. The information sources were: a) the municipal population register to estimate the population at risk, and b) the mortality register of Barcelona, which includes geocoded data for each corresponding residential address (99% of deaths) and the educational level of the deceased (93% of deaths) obtained through record linkage with the local population register.

The hierarchical dataset was yearly mortality linked-population using groups of individuals by age, sex, education (primary or less, secondary, university, unknown), country of birth (Spanish-born, foreign-born), and neighborhood of residence. Barcelona was divided into 38 neighborhoods where the population varied from 500 to 100,000 inhabitants, approximately, with a median of nearly 35,000 residents. The unemployment rate was used to characterize the socioeconomic context of the neighborhoods because it is a common and simple measure of area-based socioeconomic context widely used in research on geographical inequalities in mortality [30]. Due to the fact that the distribution of unemployment in the neighborhoods has been stable through the years, we used the unemployment rate of 2001, using quartiles (Q1 low unemployment: 7.6%-9,8%, Q2: 10–10.7%, Q3: 10.7–12.2, Q4 high unemployment 12.4–17.2%).

All analyses were stratified by sex. In addition, to estimate the effect of migration, all estimates were fitted in the total number of men and women, and separated by country of birth (Spanish- and foreign-born). Three periods were analyzed: 2001–2004, 2005–2008 and 2009–2012 (period of the financial crisis), and biannual periods were used to describe some data.

**Fig. 1:**
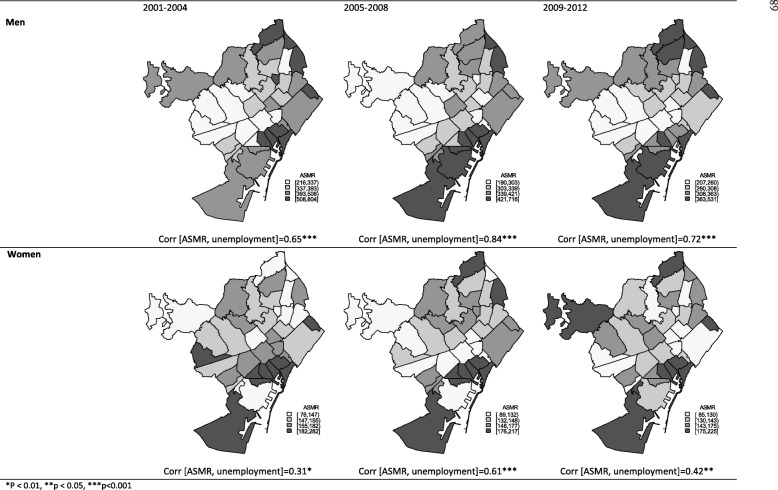
Trends in age-standardized mortality rates per 100,000 inhabitants (ASMR) in men and women aged 25–64 years in neighborhoods in Barcelona 2001–2012

First, age-standarized mortality rates per 100,000 population (ASMR) in 38 neighborhoods were estimated by period, using the direct method and the total population of Barcelona in 2001 as the reference, and ASMR were described with maps of quartiles, using time-varying ranks, in men and women (Fig. 1). Spearman correlation coefficients between unemployment and ASMR were calculated. Then, deaths and populations were obtained across neighborhoods, grouped by unemployment quartiles, and ASMRs by neighborhood unemployment groups were estimated, biannually and by period.

Finally, we applied mixed Poisson regressions using generalized linear mixed models [31], adjusted by age and education, and levels of neighborhood unemployment, to estimate the fixed effects or mortality relative risk (RR), based on Laplace Approximation, and their 95% confidence intervals, using normal approximation (95% CI). The models included two random effects to consider the intracorrelation, both within neighborhoods (spatial) and across years (temporal).

## Results

In Barcelona, the population aged 25–64 increased 8% between 2001 and 2012 (Table 1). Although the number of Spanish-born men and women decreased, the number of foreign-born residents increased, with this increase being higher in disadvantaged neighborhoods (with high unemployment rates). In these neighborhoods, in the first period (2001–2004), foreign-born residents represented 24.1% of men and 18% of women but less than 14% of residents in the rest of the city. In the second period (2005–2008), the foreign-born population increased, representing 38.1% of men and 30.4% of women residing in deprived neighborhoods, and 20% in the rest of the city. In the last period (2009–2012) the foreign-born population increased slightly, representing 42.5% of men and 36.7% of women residing in deprived neighborhoods, and almost 25% of men and women in the rest of the city.

**Table 1:** Period trends in deaths and population by neighborhood unemployment in men and women aged 25–64 years by country of birth in Barcelona 2001–2012

	Number of deaths (%)			Population (%)		
	2001–04	2005–08	2009–12	2001–04	2005–08	2009–12
Men						
Total						
Q1 Low unemployment 7.6–9.8%	1195 (100.0%)	1161 (100.0%)	1013 (100.0%)	415,382 (100.0%)	436,905 (100.0%)	441,156 (100.0%)
Q2: 10–10.7%	1252 (100.0%)	1146 (100.0%)	992 (100.0%)	371,715 (100.0%)	395,836 (100.0%)	404,034 (100.0%)
Q3: 10.7–12.2%	1995 (100.0%)	1870 (100.0%)	1672 (100.0%)	578,679 (100.0%)	615,689 (100.0%)	632,077 (100.0%)
Q4 High unemployment −12.4-17.2%	1458 (100.0%)	1313 (100.0%)	1149 (100.0%)	333,631 (100.0%)	380,819 (100.0%)	373,893 (100.0%)
Spanish-born						
Q1 Low unemployment 7.6–9.8%	1107 (92.6%)	1075 (92.6%)	929 (91.7%)	361,239 (87.0%)	344,997 (79.0%)	330,581 (74.9%)
Q2: 10–10.7%	1202 (96.0%)	1076 (93.8%)	901 (90.8%)	323,979 (87.2%)	311,058 (78.6%)	295,873 (73.2%)
Q3: 10.7–12.2%	1919 (96.2%)	1757 (94.0%)	1544 (92.3%)	511,561 (88.4%)	491,877 (79.9%)	468,396 (74.1%)
Q4 High unemployment −12.4-17.2%	1363 (93.5%)	1205 (91.8%)	1018 (88.6%)	253,210 (75.9%)	235,656 (61.9%)	214,831 (57.5%)
Foreign-born						
Q1 Low unemployment 7.6–9.8%	88 (7.4%)	86 (7.4%)	84 (8.3%)	54,143 (13.0%)	91,908 (21.0%)	110,575 (25.1%)
Q2: 10–10.7%	50 (4.0%)	71 (6.2%)	91 (9.2%)	47,736 (12.8%)	84,778 (21.4%)	108,161 (26.8%)
Q3: 10.7–12.2%	76 (3.8%)	113 (6.0%)	128 (7.7%)	67,118 (11.6%)	123,812 (20.1%)	163,681 (25.9%)
Q4 High unemployment −12.4-17.2%	95 (6.5%)	108 (8.2%)	131 (11.4%)	80,421 (24.1%)	145,163 (38.1%)	159,062 (42.5%)
Women						
Total						
Q1 Low unemployment 7.6–9.8%	701 (100.0%)	625 (100.0%)	664 (100.0%)	468,952 (100.0%)	488,479 (100.0%)	491,290 (100.0%)
Q2: 10–10.7%	594 (100.0%)	588 (100.0%)	566 (100.0%)	406,262 (100.0%)	427,504 (100.0%)	435,767 (100.0%)
Q3: 10.7–12.2%	903 (100.0%)	938 (100.0%)	888 (100.0%)	608,030 (100.0%)	641,385 (100.0%)	657,834 (100.0%)
Q4 High unemployment −12.4-17.2%	577 (100.0%)	515 (100.0%)	495 (100.0%)	304,844 (100.0%)	333,849 (100.0%)	334,953 (100.0%)
Spanish-born						
Q1 Low unemployment 7.6–9.8%	658 (93.9%)	560 (89.6%)	596 (89.8%)	404,790 (86.3%)	385,990 (79.0%)	366,927 (74.7%)
Q2: 10–10.7%	560 (94.3%)	545 (92.7%)	509 (89.9%)	354,154 (87.2%)	340,339 (79.6%)	321,210 (73.7%)
Q3: 10.7–12.2%	862 (95.5%)	880 (93.8%)	808 (91.0%)	541,249 (89.0%)	521,330 (81.3%)	494,220 (75.1%)
Q4 High unemployment −12.4-17.2%	545 (94.5%)	472 (91.7%)	428 (86.5%)	249,838 (82.0%)	232,325 (69.6%)	212,029 (63.3%)
Foreign-born						
Q1 Low unemployment 7.6–9.8%	43 (6.1%)	65 (10.4%)	68 (10.2%)	64,162 (13.7%)	102,489 (21.0%)	124,363 (25.3%)
Q2: 10–10.7%	34 (5.7%)	43 (7.3%)	57 (10.1%)	52,108 (12.8%)	87,165 (20.4%)	114,557 (26.3%)
Q3: 10.7–12.2%	41 (4.5%)	58 (6.2%)	80 (9.0%)	66,781 (11.0%)	120,055 (18.7%)	163,614 (24.9%)
Q4 High unemployment −12.4-17.2%	32 (5.5%)	43 (8.3%)	67 (13.5%)	55,006 (18.0%)	101,524 (30.4%)	122,924 (36.7%)

It is worth mentioning that the majority of foreign-born population (around 75% in the 3 periods) belongs to the 25–44 years age group, 20% to the 45–64 years age-group and 5% to the > 64 age-years group. These figures for the Spanish-born population are approximately 40%, 30%, 30%.

**Figure 2:**
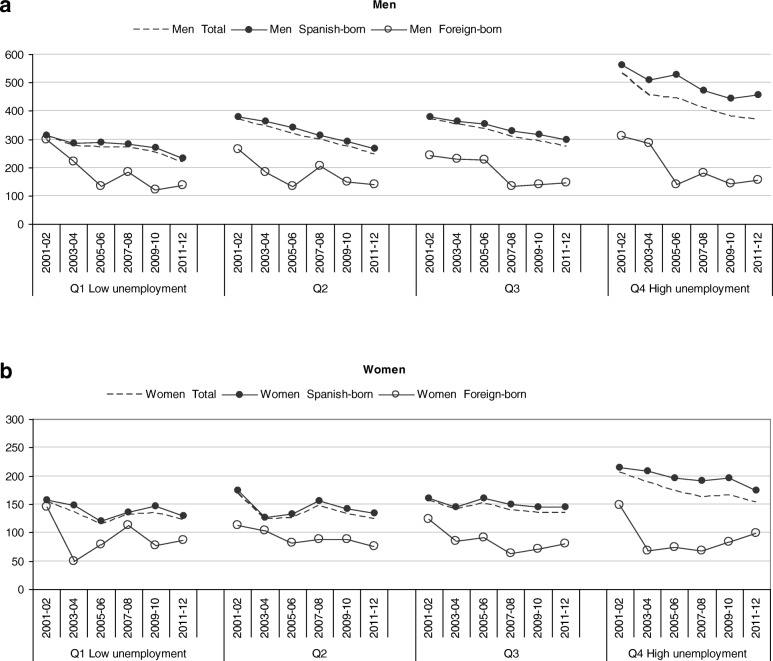
**a** Biannual trends in age-standardized mortality rates per 100,000 inhabitants (ASMR) by country of birth in men aged 25–64 years by neighborhood unemployment, Barcelona 2001–2012. **b** Biannual trends in age-standardized mortality rates per 100,000 inhabitants (ASMR) by country of birth in women aged 25–64 years by neighborhood unemployment, Barcelona 2001–2012

Between 2001 and 2012, a total of 26,148 premature deaths (25–64 years) occurred in Barcelona (67.1% men and 32.9% women), 24,270 with all socioeconomic information (92.8%). Approximately 9% of premature deaths occurred in foreign-born residents, with slight variation between neighborhoods and periods. The number of premature deaths decreased in Spanish-born men and women, but increased in the foreign-born population (Table 1). However, ASMR (Table 2) showed that premature mortality rates decreased in both the Spanish and foreign-born populations. ASMR were lower among women than men and also were lower among the foreign-born population than the Spanish-born population in both men (Fig. 2a) and women (Fig. 2b).

**Table 2:** Period trends in age-standardized mortality rates per 100,000 inhabitants (ASMR) and relative inequalities in mortality by neighborhood unemployment (RR), in men and women aged 25–64 years by country of birth, Barcelona 2001–2012

	ASMR			RR (CI95%)					
	2001–04	2005–08	2009–12	2001–04		2005–08		2009–12	
Men									
Total									
Q1 Low unemployment 7.6–9.8%	293.4	270.3	233.6	1		1		1	
Q2: 10–10.7%	356.4	308.8	260.3	1.06	(0.91–1.24)	1.00	(0.86–1.16)	0.96	(0.83–1.13)
Q3: 10.7–12.2%	359.9	322.6	282.8	1.03	(0.90–1.19)	1.00	(0.87–1.15)	1.01	(0.87–1.16)
Q4 High unemployment −12.4-17.2%	490.9	426.6	375.1	*1.40*	*(1.21–1.62)*	*1.30*	*(1.12–1.51)*	*1.33*	*(1.14–1.55)*
Spanish-born									
Q1 Low unemployment 7.6–9.8%	299.3	286.7	252.6	1		1		1	
Q2: 10–10.7%	370.3	328.6	279.7	1.08	(0.92–1.28)	0.99	(0.84–1.17)	0.94	(0.79–1.12)
Q3: 10.7–12.2%	371.9	341.5	308.2	1.05	(0.90–1.22)	0.99	(0.85–1.15)	0.99	(0.85–1.16)
Q4 High unemployment −12.4-17.2%	537.2	500.5	450.1	*1.43*	*(1.21–1.68)*	*1.34*	*(1.14–1.58)*	*1.36*	*(1.15–1.61)*
Foreign-born									
Q1 Low unemployment 7.6–9.8%	254.2	159.4	129.5	1		1		1	
Q2: 10–10.7%	215.7	171.5	144.7	0.75	(0.52–1.08)	1.01	(0.72–1.40)	1.16	(0.85–1.59)
Q3: 10.7–12.2%	235.4	176.4	143.3	0.80	(0.57–1.11)	1.11	(0.81–1.50)	1.08	(0.80–1.46)
Q4 High unemployment −12.4-17.2%	293.7	160.3	150.1	0.90	(0.65–1.25)	1.01	(0.73–1.38)	1.19	(0.87–1.61)
Women									
Total									
Q1 Low unemployment 7.6–9.8%	145.5	122.3	128.3	1		1		1	
Q2: 10–10.7%	146.4	136.9	128.2	0.95	(0.80–1.12)	1.05	(0.89–1.25)	0.94	(0.80–1.12)
Q3: 10.7–12.2%	148.3	145.7	134.0	0.94	(0.81–1.10)	1.10	(0.94–1.29)	0.97	(0.83–1.14)
Q4 High unemployment −12.4-17.2%	196.7	167.4	159.7	*1.25*	*(1.05–1.47)*	*1.27*	*(1.07–1.51)*	1.18	(0.99–1.40)
Spanish-born									
Q1 Low unemployment 7.6–9.8%	153.0	128.2	138.3	1		1		1	
Q2: 10–10.7%	150.7	144.5	138.1	0.94	(0.78–1.13)	1.08	(0.89–1.31)	0.99	(0.78–1.14)
Q3: 10.7–12.2%	152.7	155.8	145.3	0.95	(0.80–1.12)	1.14	(0.96–1.36)	0.99	(0.83–1.18)
Q4 High unemployment −12.4-17.2%	212.0	193.7	185.9	*1.28*	*(1.06–1.54)*	*1.38*	*(1.14–1.67)*	*1.23*	*(1.02–1.49)*
Foreign-born									
Q1 Low unemployment 7.6–9.8%	92.5	96.2	82.6	1		1		1	
Q2: 10–10.7%	107.7	85.0	81.0	1.16	(0.73–1.82)	0.87	(0.59–1.27)	0.95	(0.67–1.36)
Q3: 10.7–12.2%	100.3	76.7	76.1	1.08	(0.70–1.67)	0.83	(0.58–1.19)	0.91	(0.66–1.27)
Q4 High unemployment −12.4-17.2%	98.5	70.9	91.6	1.05	(0.65–1.68)	0.75	(0.50–1.11)	1.02	(0.72–1.44)

The spatial pattern observed in premature mortality was related to unemployment in the neighborhoods, in both men and women in all periods (Fig. 1). Mortality rates were higher in neighborhoods with higher unemployment rates (Spearman correlations were 0.72 and 0.42 in the period 2009–2012 in men and women, respectively).

When the data were analyzed by neighborhood unemployment groups (Table 2), premature mortality rates in men were higher in disadvantaged neighborhoods and these socioeconomic inequalities persisted across all periods. The mortality relative risk, between neighborhoods with higher and lower unemployment rates, was higher in 2001–2004 (RR = 1.40, 95%CI = 1.21–1.62), decreased in 2005–2008 (RR = 1.30, 95%CI = 1.12–1.51) and remained stable in the last period, 2009–2012 (RR = 1.33, 95%CI = 1.14–1.55. Nevertheless, socioeconomic inequalities in premature mortality were observed only in Spanish-born men (RR = 1.43, 95%CI = 1.21–1.68 in 2001–2004, RR = 1.34, 95%CI = 1.14–1.58 in 2005–2008 and RR = 1.36, 95%CI = 1.15–1.61 in 2009–2012). In foreign-born men, there was no excess mortality in disadvantaged neighborhoods., except during the last period, 2009–2012, but it was not significant. In women, premature mortality was higher in disadvantaged neighborhoods (RR = 1.25, 95%CI = 1.05–1.47 in 2001–2004 and RR = 1.27, 95%CI = 1.07–1.51 in 2005–2008), but the excess mortality was reduced in the last period and was not significant (RR = 1.18, 95%CI = 0.99–1.40 in 2009–2012). However, as in men, socioeconomic inequalities in premature mortality were observed only in Spanish-born women and also in the last period, whereas no excess mortality was observed in foreign-born women in disadvantaged neighborhoods.

## Discussion

This study has shown that premature mortality rates were higher in disadvantaged neighborhoods and in the Spanish-born population. Premature mortality decreased during the period but socioeconomic inequalities in mortality in the Spanish-born population were stable and no inequalities were found between neighborhoods in foreign-born men and women. Although socioeconomic inequalities emerged between neighborhoods in foreign born men and women in the last period, when the economic crisis began, these were non-significant among men. Evidence of the ‘healthy migrant’ effect in mortality and socioeconomic mortality inequalities was found in Barcelona, which seems to alter the distribution of mortality through time and space, related to the low levels of premature mortality. Also it is important the selective residence of immigrants in socioeconomically disadvantaged neighborhoods. Therefore, these migration effects would thus lead to false conclusions about trends in socioeoconomic inequalities in mortality unless the migrant population is taken into account.

### Socioeconomic spatial concentration of immigrants

In Barcelona, the adult population aged 25–64 years old increased in last decade mainly due to the arrival of the immigrant population, largely in socioeconomically deprived neighborhoods. This finding agrees with previous descriptions of the residential concentration patterns of immigrants in Barcelona, where the migrant population are concentrated in socioeconomically deprived areas [26]. The rapid and strong population growth has resulted in significant changes in segregation and the emergence of ethnic enclaves in Barcelona in the last decade, reflecting the diversity of nationalities and access to the housing market [7].

### Healthy migrant effect on mortality

We showed that the foreign-born population had lower levels of premature mortality than the Spanish-born population. This migrant mortality advantage has been widely studied. In Europe, many studies have revealed low levels of premature mortality among the immigrant population [9, 15–17]. The hypothesis of the “healthy migrant” effect agrees with the main characteristics of the foreign-born population in Barcelona and other metropolitan areas of Spain [6]. However, over time, this protective effect may weaken, diminishing the relative advantage of migrants over locally/born people [20]. This could explain why, in our study, excess mortality appeared in foreign-born men in socioeconomically deprived neighborhoods in the last period studied.

### Healthy migrant effect on socio-spatial inequalities in mortality

There is growing evidence of a consistent geographical pattern of socioeconomic inequalities in mortality in European cities [3]. In addition, in Spain the excess mortality found in socioeconomically disadvantaged areas has remained stable in several cities [32]. However, the present study found that in Barcelona this stabilized unequal pattern was observed only in premature mortality in the Spanish-born population, with no inequalities observed in the foreign-born population.

In addition, the greater effect of immigrants was observed in socioeconomically deprived neighborhoods, related to the higher increase in the number of migrants, in line with studies conducted in Montreal [33], and London [24] and a previous study in Barcelona [26], where socio-spatial inequalities in mortality decreased in part related to the increase in the migrant population. Our results are in line with studies based on large prospective cohorts in the Netherlands [34] and Canada [19], which found a modifying effect of immigration on area inequalities in mortality: area income inequalities were associated with mortality among the non-immigrant population only; in contrast, associations among immigrants were either absent or protective.

Although the healthy migrant effect was observed similarly in men and women, in line with other studies [13, 15–17, 34], the magnitude of neighborhood socioeconomic inequalities in premature mortality found in the Spanish population was higher in men than in women. One possible explanation lies in the specific causes of death in premature mortality in men and women [34]. In men, the main causes of premature mortality were closely related to socioeconomic position and socioeconomic context such as cardiovascular disease, lung cancer and external causes [35]; however these causes decreased in Barcelona [36, 37]. In women, the main causes of premature were cardiovascular disease and breast cancer [35], which in Barcelona held constant over time and showed no differences between socioeconomic groups [38].

As a result of this evidence, the relation between socioeconomic factors and mortality could be masked unless immigration is accounted for, and trends in excess mortality observed in socioeconomically deprived neighborhoods might be underestimated. In Barcelona, these results seem to be related to changes observed in deprived neighborhoods affecting population health: first, the arrival of immigrants and rejuvenation of the population [6] and second, the continuous processes of urban regeneration programs [39], although these changes may also produce some displacement of vulnerable populations [40].

### Economic crisis

When the current global economic crisis started in 2008, the positive trend observed in Barcelona in premature mortality and mortality inequalities seemed to start to change in some vulnerable groups [41, 42]. In Spain, due to the economic crisis, the number of employed foreigners decreased by half a million and unemployed rose to 1 million at the end of 2010. As employment fell, immigration flows also rapidly declined and the number of foreigners stopped increasing [5]. This vulnerable situation could be related to excess premature mortality among foreign-born men in socioeconomically disadvantaged neighborhoods, and among foreign-born men and women with a low educational level, supporting the hypothesis of the negative effect of the economic crisis on mortality inequalities [27, 28].

### Strengths and limitations

This study provides new evidence on trends in neighborhood socioeconomic inequalities in premature mortality in the native and foreign-born population in a southern European urban setting. To our knowledge, few studies have analyzed the effect of international immigration on trends in spatial socioeconomic inequalities in mortality**.** Some strengths of this study are the large population base, the complex data design, controlling both temporal and spatial variability and using both individual and contextual measures of socioeconomic position, and also providing specific evidence by gender.

Nonetheless, this study has some limitations. First, caution should be exercised in detecting geographic inequalities when using a repeated cross-sectional design unless selective movement of people is taken into account. In Barcelona, the intensity of residential mobility has increased over the last decade, especially as a consequence of the foreign population, which represented about half the number of residential changes registered in the city in 2008 [6]. Additionally, some studies using different information sources and based on mortality re-estimation, have revealed a two-fold underestimation of mortality among foreigners due to a biased death numerator and population denominator [43], but this underestimation does not appear to be sufficient to explain the whole mortality advantage [8, 10]. The present study used data recorded in the municipal population register and vital statistics, including all individuals who habitually live in the municipality, being a reliable source of information on the immigrant population [44].

Other limitations are the absence of second-generation immigrants, and we cannot account for the ‘salmon effect’ bias. However, we expect this bias has been attenuated because the young and older populations have been excluded, limiting our study population to persons aged 25–64 years.

Because of the small number of the foreign-born population, we did not analyze the influence of the country of origin. In Barcelona, the most frequent countries of origin were poor but with different cultural and historical backgrounds between the ethnic communities (Latin American, Arabic and Asian). Various studies have shown some differences in mortality patterns by origin, reflecting some cultural and socioeconomic determinants of migrants in the host country and country of origin [12].

## Conclusions

Our results provide evidence that migration in Barcelona is an additional factor influencing mortality dynamics. Because of varying population movements, operating at different times and locations, the effects of migration should be included in all studies examining changes in the spatial distribution of health [22]. These findings are important for evaluating public health programs. A better understanding of the processes of migration in mortality inequalities patterns could provide insights for further monitoring, action and evaluation of policies to tackle these socioeconomic inequalities.

It is important to create recognition of the positive contribution of migrants to population dynamics and urban regeneration, as this collective remains an important resource in terms of their labor and socio-cultural diversity. This requires an approach to both area regeneration and to the development of new housing, which is based on the principle of desegregation, or the creation of ‘mixed communities’ [45]. Barcelona has a long tradition of migration and social inclusion, having become well known for the social cohesion of its inhabitants. In addition, to develop and to efficiently evaluate policies to tackle health inequalities in the city, there is a need for future detailed research on population dynamics and the impact on health and health inequalities.
